# Editorial: Monocyte heterogeneity and plasticity

**DOI:** 10.3389/fimmu.2025.1601503

**Published:** 2025-04-22

**Authors:** Heather J. Medbury, Jingbo Pang, Ramalingam Bethunaickan

**Affiliations:** ^1^ Vascular Biology Research Centre, Department of Surgery, Westmead Hospital, Westmead, NSW, Australia; ^2^ Westmead Clinical School, The University of Sydney, Westmead, NSW, Australia; ^3^ Center for Wound Healing and Tissue Regeneration, Department of Kinesiology and Nutrition, University of Illinois at Chicago, Chicago, IL, United States; ^4^ Department of Immunology, Indian Council of Medical Research (ICMR)-National Institute for Research in Tuberculosis, Chennai, India; ^5^ Department of Immunology, University of Madras, Chennai, India; ^6^ Faculty of Medical Research, Academy of Scientific and Innovative Research (AcSIR), Ghaziabad, India

**Keywords:** monocyte, monocyte heterogeneity, monocyte plasticity, monocyte reprogramming, monocyte subset

Monocytes are innate immune cells that are formed in the bone marrow before being released into the blood (1). They differentiate in the circulation with three major monocyte subsets identified in humans: classical, intermediate and non-classical (2). While the subsets show phenotypic and functional differences (3–5), there is also considerable heterogeneity within each subset (6). In addition, monocytes exhibit plasticity, responding to factors in their environment (7). These functional modifications highlight the ability of monocytes to play a unique role in homeostasis and disease, even before their migration and differentiation in the tissues.

Notably, monocyte precursors (in the bone marrow) can also respond directly to their environment, predetermining the basal state of an individual’s monocytes (8). The degree to which these functional modifications are recapitulated during differentiation, or whether they enable the cell to mount a trained response upon exposure to other stimuli, is a growing area of investigation.

Here we gathered the latest insights on monocyte classification, heterogeneity, function and plasticity in homeostasis and across different diseases.

Recent research highlights the significance of monocyte heterogeneity in the development and progression of many diseases, suggesting some intriguing and promising approaches for future diagnosis and treatment of many pathological conditions. Among these diseases, Chen et al. reviewed the current understanding and updates of monocyte heterogeneity in the pathogenesis of coronary artery disease (CAD), including several types of both stable and acute CADs. The levels of three subsets of monocytes, classical (CD14++CD16−), intermediate (CD14++CD16+), and non-classical (CD14+CD16++) were reported using the more recent terminology for these subsets: Mon1, Mon2 and Mon3, respectively. They also looked at the pro- or anti-inflammatory cytokines, associated with these subsets. While all studies reported dramatic changes in CAD patients compared to the healthy controls, there were some inconsistent reports regarding the ratios of Mon1, Mon2 and Mon3 in some patients. This may be due to sample size, geographic variability, types of CAD, and more importantly, the high complexity of monocyte heterogeneity. The attempt to simply classify monocytes into Mon1, Mon2, and Mon3 categories based on the expression of a small number of markers may not reflect the true heterogeneity of monocytes in CAD. The utilization of more advanced and comprehensive experimental approaches, such as spectral flow cytometry and single-cell RNA sequencing, would further elucidate the complexity of monocyte heterogeneity, and their functions in CAD.

That monocytes are heterogeneous is further supported by the work of Ruiz et al., who examined sub-populations within human CD14+CD16+ monocytes matured *in vitro*. Of the 9 distinct clusters identified, 3 had increased gene expression for migratory and inflammatory pathways. The authors propose that these clusters, as a group, may play a role in neuroinflammatory conditions as they functionally produce higher levels of cytokines and preferentially transmigrate in an *in vitro* blood-brain barrier model. Although the cells had been matured *in vitro* from classical monocytes, the study adds further weight to the understanding that monocytes are highly heterogeneous, with some subgroups better equipped to play an active role in certain disease states.

In line with this, Chen et al. found that the proportion of CD16+ monocytes is increased in patients with chronic thromboembolic pulmonary hypertension (CTEPH) and that this subset was enriched in genes that contribute to T cell activation, coagulation and platelet activation; functions that are important in CTEPH. They propose that CD16+ monocytes may function as a biomarker to support imaging results in the diagnosis of CTEPH - which would be much less invasive than procedures recommended by current guidelines. Increased numbers of different monocyte subsets are associated with different diseases (9), so the elevated levels alone do not play a definitive role. There would also need to be clear standardization of monocyte gating steps for this to be adopted across different centers. Chen et al. also provided insight into how the CD16+ monocytes may contribute to CTEPH, with the increased migratory phenotype, compared to that of monocytes from controls, enabling them to migrate into tissues. Thus, akin to macrophages, there is not only great heterogeneity of monocytes, but also plasticity as seen by their phenotypic changes in disease.

Phenotypic changes can occur by reprogramming and Chagovets et al. (focusing on CD14+ monocytes) demonstrated tumor-induced reprogramming of amino acid metabolism in peripheral blood monocytes across various cancer types. The authors identified distinct amino acid profiles in circulating monocytes from patients with different tumors, including breast, ovarian, lung, and colorectal cancer. Notably, they observed unique amino acid signatures specific to each cancer type. These immune metabolomic alterations may result from the prolonged influence of tumor cells originating from different anatomical sites. The study explores the connection between amino acid metabolism in monocytes and their functional dichotomy, which is governed by the pro- and anti-inflammatory activity of macrophages. This, in turn, contributes to the programming of tumor-associated macrophages (TAMs). Significant metabolic changes were noted, including elevated levels of tryptophan in ovarian cancer, aminobutyric acid in lung cancer, aspartic acid in colorectal cancer, and sarcosine and glutamic acid in ovarian cancer. Additionally, the study highlights alterations in citrullination that may serve as markers for differentiation between cancer types. Overall, this research provides valuable insights into monocyte metabolism and the transcriptional and epigenetic modifications that contribute to the heterogeneity of TAM programming. These findings are crucial for the development of targeted therapeutic strategies aimed at modulating TAM activity in cancer treatment.

The plasticity – or re-education- of monocytes was further demonstrated in the study by Larionova et. al., where monocytes in patients with ovarian cancer displayed decreased inflammation and antigen presenting ability. Interestingly, however, chemotherapy had a greater effect on the monocyte transcriptome, than the cancer status of the studied subjects. One key feature was increased antigen presentation ability by transcriptional upregulation of MHC class II molecules. However, this was not due to epigenetic changes.

Collectively, the articles in this Research Topic provide further insight into the complexity of monocytes and how they exhibit remarkable heterogeneity and phenotypic flexibility that impact a wide range of diseases and conditions. The fact that changes occur in monocytes, prior to their entry into the tissues and differentiation into macrophages further highlights the importance of this field. There is a great need for further studies to explore this topic, particularly under different disease conditions. Moreover, doing so using animal models will be important as this will also make it easier to examine the impact of monocyte changes on the function of their (migrated and differentiated) macrophage form.
